# Continuous exposure to the deterrents *cis*-jasmone and methyl jasmonate does not alter the behavioural responses of *Frankliniella occidentalis*

**DOI:** 10.1111/eea.12381

**Published:** 2015-12-12

**Authors:** Barbara Egger, Bernhard Spangl, Elisabeth Helene Koschier

**Affiliations:** 1 Division of Plant Protection, Department of Crop Sciences, University of Natural Resources and Life Sciences (BOKU)Peter-Jordan-Straße 82, 1190, Vienna, Austria; 2 Institute of Applied Statistics and Computing (IASC), Department of Landscape, Spatial and Infrastructure Sciences, University of Natural Resources and Life Sciences (BOKU)Peter-Jordan-Straße 82, 1190, Vienna, Austria

**Keywords:** western flower thrips, secondary plant compound, feeding deterrence, habituation, Thysanoptera, Thripidae

## Abstract

Behavioural responses of *Frankliniella occidentalis* (Pergande) (Thysanoptera: Thripidae), a generalist, cell sap-feeding insect species with piercing-sucking mouthparts, after continuous exposure to two deterrent secondary plant compounds are investigated. We compared in choice assays on bean leaf discs, the settling, feeding, and oviposition preferences of *F. occidentalis* females that had no experience with the two fatty acid derivatives methyl jasmonate and *cis*-jasmone before testing (naïve thrips) vs. females that had been exposed to the deterrent compounds before testing (experienced thrips). The thrips were exposed to the deterrents at low or high concentrations for varied time periods and subsequently tested on bean leaf discs treated with the respective deterrent at either a low or a high concentration. *Frankliniella occidentalis* females avoided settling on the deterrent-treated bean leaf discs for an observation period of 6 h, independent of their previous experience. Our results demonstrate that feeding and oviposition deterrence of the jasmonates to the thrips were not altered by continuous exposure of the thrips to the jasmonates. Habituation was not induced, neither by exposure to the low concentration of the deterrents nor by exposure to the high concentration. These results indicate that the risk of habituation to two volatile deterrent compounds after repeated exposure is not evident in *F. occidentalis*. This makes the two compounds potential candidates to be integrated in pest management strategies.

## Introduction

*Frankliniella occidentalis* (Pergande) (Thysanoptera: Thripidae), the western flower thrips, is a major pest on many horticultural and agricultural crops worldwide (Kirk & Terry, 2003). These thrips feed on epidermal and mesophyll cells by penetrating them with their piercing-sucking mouthparts (Lewis, 1973; Childers, 1997). Being a potent vector of plant viruses, *F. occidentalis* mediate additional damage to plants (Wijkamp et al., 1995). Management of *F. occidentalis* is problematic due to their minute size and their thigmotactic behaviour (Lewis, 1997). Furthermore, the repeated use of chemical insecticides resulted in widespread development of resistance in *F. occidentalis* (Jensen, 2000). Behavioural manipulation strategies could offer possible alternative approaches to thrips control. Disrupting the host plant acceptance behaviour of thrips by using secondary plant compounds that act as feeding and oviposition deterrents is considered to have great potential (Cowles, 2004; Cook et al., 2007).

*Cis*-jasmone and methyl jasmonate are on one hand constitutively present as compounds of essential oils in some plant species (e.g., *Jasminum* spp., *Lonicera* spp., or *Philadelphus* spp.). On the other hand, being stress-related secondary plant compounds, both are known to play a major role in plant defence mechanisms against herbivores (Joulain, 1986; Mookherjee et al., 1990; Birkett et al., 2000; Howe & Jander, 2008).

In various laboratory and field studies, exogenous applications of jasmonates to plants resulted in the induction of plant resistance to herbivores. Various aphid species (Thaler et al., 2001; Bruce et al., 2003a,b; Glinwood et al., 2007; Brunissen et al., 2010), the two-spotted spider mite *Tetranychus urticae* Koch (Rohwer & Erwin, 2010), and *F. occidentalis* (Thaler et al., 2001) avoid jasmonate-treated plants or plant parts. Furthermore, methyl jasmonate and *cis*-jasmone may act as an indirect defence mechanism of plants by attracting natural enemies of the herbivores, among them the aphid parasitoid *Aphidius ervi* Haliday and other hymenopterous parasitoids (Bruce et al., 2003b; Simpson et al., 2011).

Direct repellent effects of *cis*-jasmone on the grain aphid, *Sitobion avenae* Fabricius, and the lettuce aphid, *Nasonovia ribis-nigri* Mosley, were revealed in olfactometer studies (Bruce et al., 2003a,b). Furthermore, both methyl jasmonate and *cis*-jasmone deterred feeding and oviposition of *F. occidentalis* adults and larvae when applied on bean leaf discs (Egger & Koschier, 2014; Egger et al., 2014).

Habituation is the waning of a response as a result of repeated presentation of a stimulus (Chapman & Bernays, 1989; Schoonhoven et al., 2005). This experience-based response may reduce the feeding-deterrent effect of secondary compounds, and thus limit their practical applicability (Jermy et al., 1982; Jermy, 1987; Glendinning & Gonzalez, 1995; Akhtar & Isman, 2003). Habituation may occur more frequently in polyphagous species such as *F. occidentalis* presumably because they have evolved mechanisms for dealing with plant defensive compounds (Bernays & Chapman, 1994; Bernays et al., 2000). Nymphs of the polyphagous locust *Schistocerca gregaria* (Forskål) and larvae of the polyphagous lepidopteran *Mamestra brassicae* (L.) habituated to feeding deterrents (Jermy et al., 1982). The intensity of the deterrent stimulus presented to the herbivores has an impact on the habituation potential: weak stimuli induce habituation, whereas strong stimuli do not (Jermy et al., 1982; Szentesi & Bernays, 1984). In a previous study, *F. occidentalis* females were repeatedly exposed to the deterrent compounds methyl jasmonate and *cis*-jasmone. Exposures for 6-h periods over four consecutive days to either jasmonate at low concentrations resulted in habituation of the thrips, whereas exposure to high concentrations did not (Egger et al., 2014).

To test whether longer exposure periods to deterrents at different concentrations induce habituation in thrips, we study here the effect of continuous exposure of at least 48 h on the responses of adult female *F. occidentalis* to two deterrents. We designed bioassays where thrips are exposed to deterrents at varying concentrations and exposure periods to account for various potential habituation scenarios. We compare the settling behaviour, the feeding preference, and the oviposition preference of young adult thrips that had no experience with the deterrents before testing (naïve thrips) vs. thrips that were exposed to the deterrent compounds before testing (experienced thrips) in choice assays on bean leaf discs.

## Materials and methods

### Insects and plants

*Frankliniella occidentalis* were collected from ornamental plants in the experimental greenhouse of the University of Natural Resources and Life Sciences in Vienna, Austria. The thrips were maintained in a laboratory on detached bean leaves [*Phaseolus vulgaris* L. cv. Borlotto (Fabaceae); Austrosaat, Vienna, Austria] on 1% (wt/vol) water agar (Sigma-Aldrich, Vienna, Austria) in plastic Petri dishes (14 cm diameter) in a climate chamber at 24 ± 1 °C, 35 ± 5% r.h., and L16:D8 photoperiod. About 50 adult females were allowed to lay eggs on bean leaves in the Petri dishes. The dishes were closed with lids with central holes covered with a fine mesh to ensure ventilation. After 48 h, the thrips were removed and the leaves with eggs were kept in Petri dishes in the climate chamber until adults emerged.

To obtain groups of even-aged thrips females for the bioassays, thrips pupae were collected from the rearing containers and transferred to fresh bean leaves on 1% water agar in separate Petri dishes. Females were tested 48 h after adult emergence, i.e., at the end of their pre-oviposition period (van Rijn et al., 1995), at the earliest. Bean plants used for rearing as well as for testing were grown in a plant-growing room at 25 ± 1 °C, 50 ± 5% r.h., and L16:D8 photoperiod in groups of 13–15 plants per pot. Leaf discs used for the bioassays were punched with a cork borer (1.1 cm diameter) from cotyledons of bean plants 11–13 days after sowing.

### Test compounds

*Cis*-jasmone and methyl jasmonate were purchased from Sigma-Aldrich. Pure compounds were diluted in pure ethanol (96%; Merck, Darmstadt, Germany) at a ratio of 1:10 (vol/vol). Subsequently, distilled water plus Triton X-100 (0.05% vol/vol; Sigma-Aldrich) as surfactant was added to obtain 15 and 50% feeding deterrent concentrations (FDC_15_ and FDC_50_), required to reduce feeding damage on the treated leaf disc by 15 or 50%, respectively, compared to control leaf disc in choice assays (Egger et al., 2014). For *cis*-jasmone, FDC_15_ was about 0.29% (hereafter referred to as ‘low concentration’) and FDC_50_ was about 0.66% (‘high concentration’); for methyl jasmonate, the low concentration was at about 0.08%, the high concentration at about 0.77%. The control solution consisted of ethanol and distilled water with the surfactant at a ratio of 1:10.

### General bioassay procedure

For the leaf disc bioassays, the bean leaf discs were put in a glass Petri dish (9 cm diameter) and sprayed with the respective dilution using a Potter Precision Laboratory Spray Tower (Burkard Manufacturing, Rickmansworth, UK) at constant air pressure resulting in an exact wet deposit of 1 μl cm^−2^ dilution quantity on the upper surface of each leaf disc. Before releasing the test insects, the treated leaf discs were allowed to dry for ca. 10 min, then placed on a 1% water agar layer in glass Petri dishes to prevent the leaf discs from wilting and to prevent the thrips from feeding on the lower leaf surfaces. The Petri dishes were closed with a plastic sealing film (Carl Roth, Karlsruhe, Germany), which was subsequently perforated by means of insect pins (ca. 10 punctures cm^−2^) to ensure ventilation, and placed in a climate chamber at 24 ± 1 °C, 35 ± 5% r.h., and L16:D8 photoperiod.

### Long-term, continuous exposure

Bean leaf discs (2.1 cm diameter) were sprayed with the low or high concentration of the test compounds or the control solution. Subsequently, 3–4 treated leaf discs were placed in a plastic Petri dish (9 cm diameter) on a thin water agar layer. About 30–40 thrips females were placed in each Petri dish on the day of their emergence from the pupal stage using a fine brush, the dishes were closed with lids with central holes covered with fine mesh.

**Figure 1:**
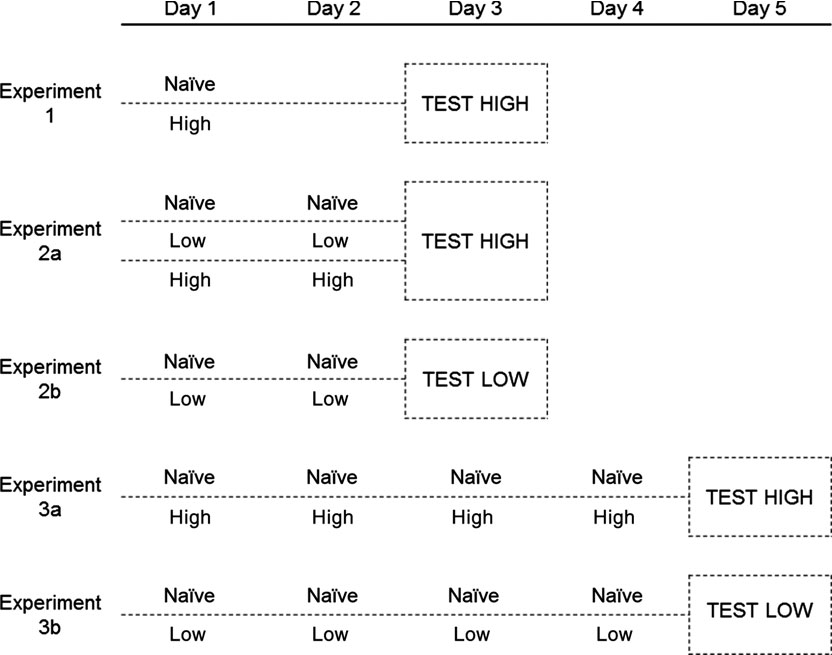
Experimental setup: *Frankliniella occidentalis* were exposed to *cis*-jasmone, methyl jasmonate, or the control solution before testing. Experiment 1: at the beginning of the exposure period, the thrips females were transferred once to leaf discs freshly sprayed with test compound at high concentration and kept in the dishes for 48 h (one application, 48 h exposure). Experiment 2a+b: at the beginning of the exposure period, thrips females were transferred to leaf discs freshly sprayed with test compound at low or high concentration and kept for 24 h, subsequently the females were transferred to freshly sprayed leaf discs and kept for another 24 h (two applications, 48 h exposure). Experiment 3a+b: as experiment 2, but the procedure was repeated two more times (four applications, 96 h exposure) prior to testing females in the choice assays.

Thrips were kept in the Petri dishes with leaf discs treated with a deterrent compound (experienced thrips) or with control leaf discs (naïve thrips) during three exposure periods (Figure1). In experiment 1, at the beginning of the exposure period the thrips females were transferred once to leaf discs freshly sprayed with one of the test compounds at high concentration, or with the control solution, and kept in the dishes for 48 h (one application, 48 h exposure). In experiments 2a and 2b, at the beginning of the exposure period thrips females were transferred to leaf discs freshly sprayed with one of the test compounds at low or high concentration, or the control solution, and kept in the dishes for 24 h, then the females were transferred to freshly sprayed leaf discs and kept there for another 24 h (two applications, 48 h exposure). In experiments 3a and 3b, the latter procedure was executed 4× (four applications, 96 h exposure) prior to testing females in the choice assays.

### Settling preference

In a choice assay, the effect of the continuous long-term exposure of 48 h (experiment 1) on the settling preference of single *F. occidentalis* females was monitored for a 6-h period. Females were exposed to high concentration *cis*-jasmone or methyl jasmonate, or control solution, on the day of their emergence from pupal stage. After 48 h, they were transferred to a Petri dish (6 cm diameter) with two leaf discs (1.1 cm diameter) ca. 4 cm apart, one treated with control solution, the other treated with a test compound. The thrips female was released in the centre of the dish. After 30, 60, 120, 180, 240, 300, and 360 min, the position of the thrips on either leaf disc or elsewhere in the Petri dish was recorded. The assay was replicated with 25–30 females per treatment.

### Feeding and oviposition preference

In a choice assay, the effect of the continuous long-term exposure periods on the feeding preference of single *F. occidentalis* females was tested. At the end of an exposure period, as described above, thrips females were transferred to Petri dishes (6 cm diameter) with two leaf discs (1.1 cm diameter) at 4 cm apart, one treated with control solution, the other treated with a test solution. The dishes were sealed with perforated plastic sealing film and kept in a climate chamber as described above. After 24 h, the females were removed. The area of feeding damage on each leaf disc was measured using a transparent counting grid (0.25 × 0.25 mm; Boraident, Halle, Germany) and a stereo microscope (Stemi 2000; Zeiss, Vienna, Austria). A feeding deterrence index (FDI) was calculated using the formula

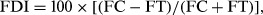
where FC and FT are the control and treated leaf areas damaged by the *F. occidentalis* females, respectively (Isman et al., 1990). The oviposition preference was measured by counting the eggs laid on the leaf discs during the 24-h test period using a stereomicroscope with transmitting light and the oviposition deterrence index (ODI) was calculated in analogy to the FDI. The assay was replicated with 24–31 females per treatment.

### Statistical analysis

The settling preference was analysed by a Generalized Linear Mixed Model (GLMM) to estimate the effect of the factors habituation, treatment, their interaction, and the covariate time. The subjects were considered as random factor to account for repeated measurements. The FDI and ODI were analysed using the Welch’s t-test or repeated measures ANOVA, separating the means by Tukey post-hoc tests. Feeding deterrence and oviposition deterrence were compared by a Pearson’s correlation. All statistical analyses were performed using the statistical package R 2.15.2 (http://www.R-project.org).

## Results

### Settling preference

**Figure 2:**
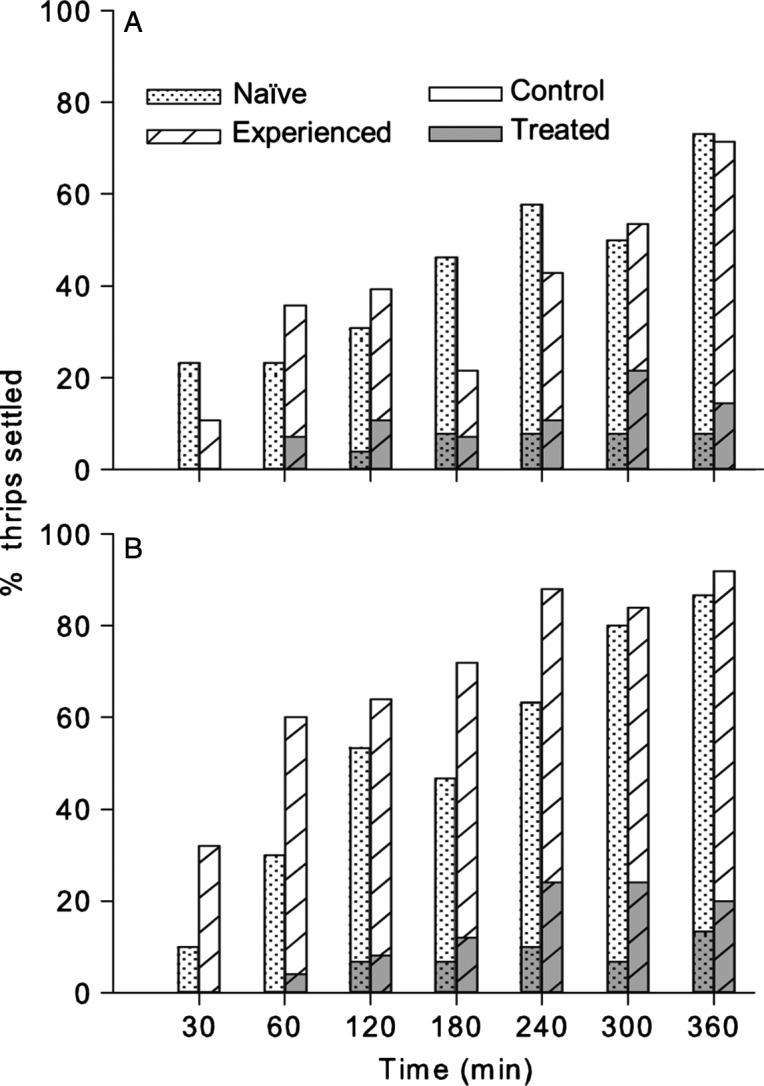
Settling preference (%) of naïve or experienced *Frankliniella occidentalis* females on (A) *cis*-jasmone-treated, (B) methyl jasmonate-treated, or control leaf discs in a dual choice assay over a 6-h observation period (n = 25–30 females per exposure duration × treatment combination).

The percentage of thrips females settling on the *cis*-jasmone- and methyl jasmonate-treated and the control leaf disc increased over the 6-h observation period, both for the naïve and for the experienced thrips (Figure2). Independent of their experience, 80–100% of the thrips avoided settling on the deterrent-treated leaf discs over the 6-h observation period. The analysis of deviance indicated that time affected settling significantly, whereas there were no significant effects of treatment compound or experience (Table1).

**Table 1:** Analysis of deviance of the settling preference of *Frankliniella occidentalis*

Factor	χ^2^	d.f.	P
Treatment	0.1088	1	0.74
Habituation	2.4028	1	0.12
Time	99.6724	1	<0.001
Treatment^*^habituation	0.0734	1	0.79

n = 25–30 thrips females per exposure duration × treatment combination.

Data were analysed by a Generalized Linear Mixed Model (GLMM).

### Feeding and oviposition preference

Feeding and oviposition deterrence were strongly positively correlated (Pearson’s correlation: r = 0.89, d.f. = 626, P<0.001).

#### Experiment 1

**Figure 3:**
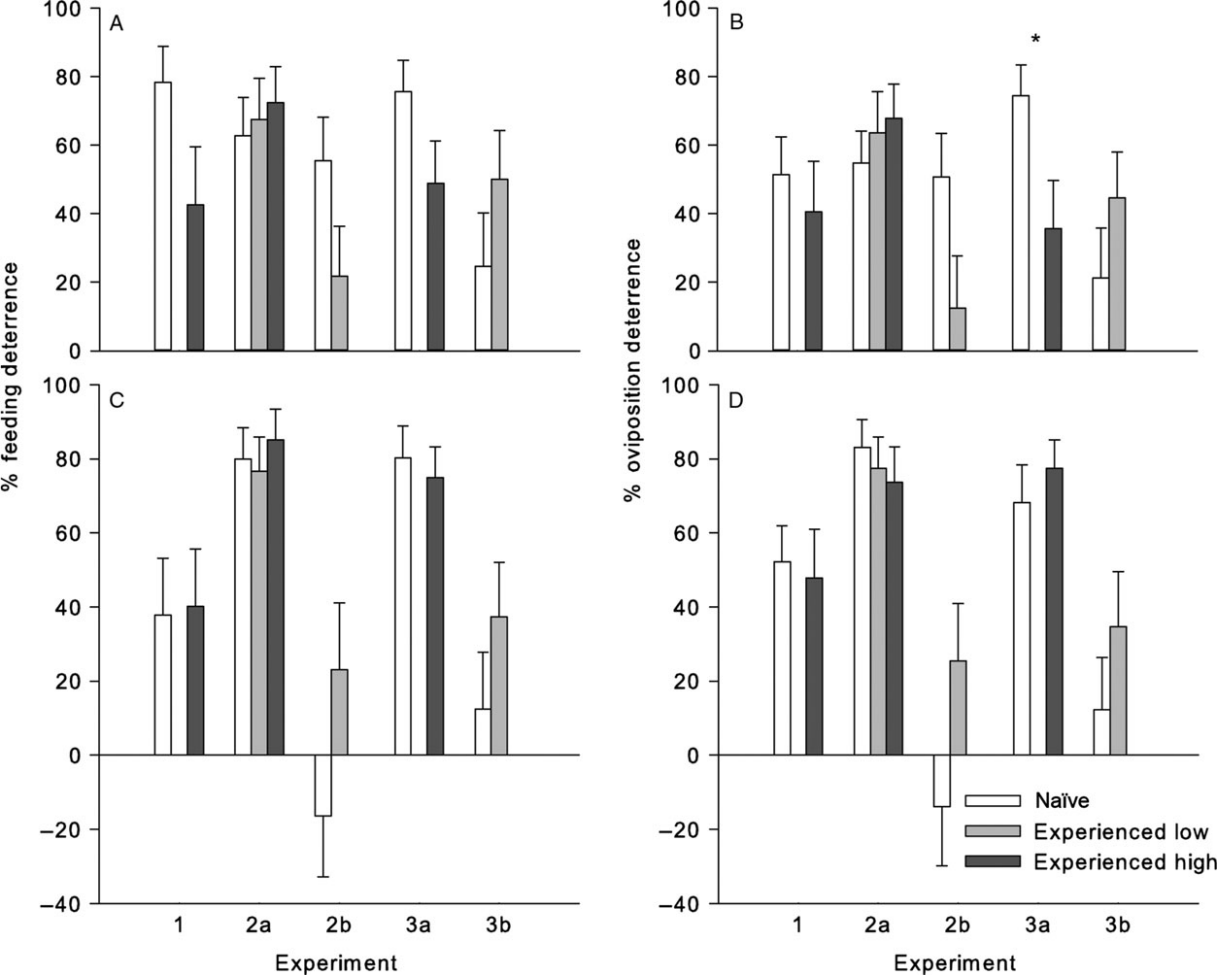
Mean (+ SEM) (A, C) feeding and (B, D) oviposition deterrence of (A, B) *cis*-jasmone and (C, D) methyl jasmonate to *Frankliniella occidentalis* females after various exposure periods (n = 24–31 thrips females per exposure duration × treatment combination). *Indicates significant difference (Welsh’s t-test: P<0.05).

Experience of adult *F. occidentalis* females with *cis*-jasmone at the high concentration did not affect their feeding or oviposition preference (Table2). When tested on leaf discs treated with the high *cis*-jasmone concentration, both the naïve and the experienced thrips preferred the control leaf discs for feeding. The FDI for *cis*-jasmone was 78% for naïve and 43% for experienced thrips (Figure3A). The ODI was 51% for naïve and 40% for experienced thrips (Figure3B). Similarly, naïve and experienced thrips were deterred by methyl jasmonate at its high concentration – experience with the deterrent did not affect their feeding or oviposition preference (Table2). The FDI for methyl jasmonate was 38% for naïve and 40% for experienced thrips (Figure3C). The ODI was 52% for naïve and 48% for experienced thrips (Figure3D).

**Table 2:** Analysis of variance of feeding and oviposition deterrence of *Frankliniella occidentalis*

Experiment	Treatment	Feeding deterrence			Oviposition deterrence		
		d.f.	F	P	d.f.	F	P
1	*Cis*-jasmone	1,45.74	3.37	>0.05	1,49.62	0.316	>0.05
	Methyl jasmonate	1,53	0.011	>0.05	1,53	0.077	>0.05
2a	*Cis*-jasmone	2,72	0.173	>0.05	2,72	0.375	>0.05
	Methyl jasmonate	2,80	0.25	>0.05	2,80	0.301	>0.05
2b	*Cis*-jasmone	1,58	3.037	>0.05	1,58	3.753	>0.05
	Methyl jasmonate	1,49	2.612	>0.05	1,49	3.103	>0.05
3a	*Cis*-jasmone	1,50.99	3.009	>0.05	1,46.86	5.356	0.025
	Methyl jasmonate	1,56	0.204	>0.05	1,56	0.534	>0.05
3b	*Cis*-jasmone	1,52	1.438	>0.05	1,52	1.402	>0.05
	Methyl jasmonate	1,52	1.354	>0.05	1,52	1.202	>0.05

24–31 thrips females were tested per exposure duration × treatment combination.

Data were analysed by a Welch’s t-test or repeated measures ANOVA, separating the means by Tukey post-hoc tests.

#### Experiment 2

Experience with either the low or the high concentration of *cis*-jasmone did not affect the feeding and oviposition preference of the thrips when tested on leaf discs treated with the low concentration of *cis*-jasmone, nor on leaf discs treated with high concentration of *cis*-jasmone (Table2). In experiment 2a, the FDI calculated for naïve thrips tested on leaf discs treated with the high *cis*-jasmone concentration was 63% (ODI 55%), for thrips with experience with the low *cis*-jasmone concentration and tested on the high concentration, the FDI was 67% (ODI 63%), and for thrips with experience with the high concentration and tested on the high concentration, the FDI was 72% (ODI 67%). Feeding of naïve thrips tested on the low *cis*-jasmone concentration was deterred by 55%, oviposition was deterred by 51%; the feeding of thrips with experience with the low *cis*-jasmone concentration and tested on the low concentration was deterred by 22%, oviposition was deterred by 12% (experiment 2b, Figure3A and B).

Likewise, experience with either the low or the high concentration of methyl jasmonate did not affect the feeding and oviposition preference of the thrips when tested on leaf discs treated with either low or high concentration of methyl jasmonate (Table2). In experiment 2a, the FDI calculated for the naïve thrips tested on the high methyl jasmonate concentration was 80% (ODI 83%), for thrips with experience with the low methyl jasmonate concentration and tested on the high concentration, the FDI was 77% (ODI 77%); for thrips with experience with the high concentration and tested on the high concentration, the FDI was 85% (ODI 74%). The FDI calculated for naïve thrips tested on the low methyl jasmonate concentration was −16% (ODI −14%), whereas thrips with experience with the low concentration of methyl jasmonate and tested on the low concentration were deterred by 23% (ODI 25%; experiment 2b, Figure3C and D).

#### Experiment 3

Experience of thrips with either the low or the high concentration of *cis*-jasmone did not affect their feeding preference (Table2). There was a significant difference in the oviposition deterrence when the thrips had been exposed previously to the high concentration and then tested on the high concentration of *cis*-jasmone (experiment 3a, Table2). Experienced thrips were less deterred than naïve thrips. In experiment 3a, the FDI calculated for the naïve thrips tested on the high *cis*-jasmone concentration was 76% (ODI 74%), whereas thrips with experience with the high *cis*-jasmone concentration were deterred by 49% (ODI 36%). Naïve thrips tested on the low *cis*-jasmone concentration (experiment 3b) were deterred by 25% (ODI 21%), the experienced thrips were deterred by about 50% (ODI 45%; Figure3A and B).

Naïve and experienced thrips were deterred by methyl jasmonate at its low and high concentration – experience with the deterrent did not affect the feeding preference (Table2). The FDI calculated for the naïve thrips tested on the high methyl jasmonate concentration (experiment 3a) was 80% (ODI 68%) and for the experienced thrips 75% (ODI 77%). In experiment 3b, feeding and oviposition of naïve thrips were both deterred by 12%, by the low methyl jasmonate concentration. The feeding of experienced thrips was deterred by 37%, oviposition by 35% (Figure3C and D).

## Discussion

*Cis*-jasmone and methyl jasmonate deter *F. occidentalis* females from feeding (Egger et al., 2014). We could confirm the deterrent effect of the two jasmonate derivatives in this study. The choice assays with the deterrents applied to bean leaf discs at their FDC_50_ caused about 50% of the naïve thrips females to avoid feeding and egg laying on the treated leaf disc. Naïve thrips also avoided settling on jasmonate-treated leaf discs over a 6-h observation period. But even a 48-h exposure period of early-adult thrips females during their pre-oviposition period did not influence their avoidance of the treated leaf discs.

In some cases, the application of the deterrents at their low or high concentrations (FDC_15_ or FDC_50_) deterred even more than 15 or 50% of the thrips from feeding, owing to the fact that the FDC values are based on mean values. For the same reason, the measured feeding or oviposition deterrence will occasionally be somewhat lower than expected at the applied concentration – in our study, this was the case in two experiments. When methyl jasmonate was used at the FDC_15_ in experiment 2b, we observed a trend to decreased deterrence effects in naïve thrips. Although this was not statistically significant, it indicates that methyl jasmonate at its low concentration may be too weak a deterrent stimulus to be a stable deterrent agent against *F. occidentalis*. A similar reduction in deterrence could be observed when methyl jasmonate at a low concentration was used as compound in binary mixtures against thrips (Egger et al., 2014). In experiment 3a, the oviposition deterrent effect differed statistically between naïve and experienced thrips: the effect decreased in experienced thrips. This could be interpreted as a decrease in responsiveness to the deterrent. However, considering that this effect was observed only in one experiment, this variation might be explained more likely by the high natural variability in *F. occidentalis* populations (de Kogel et al., 1998).

For the potential future implementation of deterrents in thrips control strategies, it is important to investigate the responses of young adult female *F. occidentalis*, because they are the host-seeking instar and young females lay most of their eggs in the 5-day period after emergence (van Rijn et al., 1995). This study covers the little-investigated responses of adult females to feeding and oviposition deterrents: compared with the amount of data collected on experience-induced behavioural changes associated with feeding by larvae, for instance of acridid or lepidopteran species (e.g., Glendinning & Gonzalez, 1995; Akhtar & Isman, 2003, 2004), much less is known about the mechanisms of changes in host selection and acceptance in adult insects (Schoonhoven et al., 2005), although they are often the host-seeking instar. Only few studies have examined habituation responses to feeding or oviposition deterrents of coleopteran (Held et al., 2001; Akhtar & Isman, 2004), lepidopteran (Wang et al., 2008), or thysanopteran (Egger et al., 2014) adult instars.

As thrips are weak flyers (Lewis, 1973), landing in a crop field treated with deterrents presumably implies they get in contact with the deterrents repeatedly and remain exposed to the substances for several days. Our experimental design allowed us to detect alterations in responsiveness of the thrips to the deterrents not only after various exposure periods but also at different deterrent concentrations. As the tested deterrent compounds are volatile, the thrips were transferred repeatedly to freshly treated plant material to ensure a constant contact of the thrips to the deterrent compounds.

The exposure to low- and high-concentrated deterrents could help to evaluate the hypothesis that weak stimuli are likely to induce habituation whereas strong stimuli do not (Jermy et al., 1982; Szentesi & Bernays, 1984). In a previous study, weakly deterrent stimuli induced habituation in thrips, whereas strong stimuli did not. The thrips then were exposed repeatedly for short time periods to the deterrent (6 h per day on four consecutive days) in a no-choice bioassay (Egger et al., 2014). In the present study, thrips were continuously exposed for longer periods to account for their habituation potential. Although in this study, the exposure periods were of up to 96 h, the deterrent effect of the jasmonates to the thrips was not reduced. The exposure neither to the low concentration nor to the high concentration of the deterrents induced habituation. This may be explained by the choice-situation: when the thrips have the choice between deterrent-treated and control leaf discs, they prefer the control leaf disc for feeding.

Our results show that no risk of habituation to two volatile deterrent compounds after continuous exposure is present in *F. occidentalis*. The reduced responsiveness of the thrips to the low-concentrated deterrents observed in the previous study was not corroborated in the present study when they were exposed continuously to the deterrents. The deterrent effect of *cis*-jasmone and methyl jasmonate was not reduced by continuous exposure of the thrips neither to the low concentration nor to the high concentration of the jasmonates. This makes the two compounds potential candidates to be integrated in pest management strategies. In this study, habituation effects to deterrents in thrips were examined in choice assays, a situation that may not be representative for crops cultivated in monocultural systems, although in a sprayed crop there may always be unsprayed spots that may act as refuges. In the next step, our findings need to be verified in no-choice bioassays. On the other hand, deterrents might be deployed most successfully in association with attractants in push–pull strategies. Their application to crop plants elicits thrips movement until they encounter the attractive trap-crop, where they can be controlled by either a biological control agent or an effective botanical. This offers an alternative to the use of synthetic insecticides.

Considering that methyl jasmonate and *cis*-jasmone showed to be not only deterrent to *F. occidentalis* but also attractive to some hymenopterous parasitoids of thrips, the two jasmonates are of potential use as synergists in biological plant control programmes (Thaler, 1999; James, 2005; James & Grasswitz, 2005; Simpson et al., 2011).

In this study, the direct effects of the applied jasmonates on thrips were investigated. A potential indirect effect on the cut bean leaf discs resulting from the application of the jasmonates or the cutting of the leaves cannot be completely excluded, although it is not very likely considering that not even cutting of leaf discs induced the emission of volatiles in a recent study (Wei et al., 2014). This supports the conclusion that thrips behaviour is influenced by the applied deterrents, not by jasmonate-induced effects.

The benefit of exogenous application of methyl jasmonate to different plants was reported to induce a higher resistance level against herbivores, but the continuous exogenous induction of plant defence may be costly. Potential negative effects on growth rate, size, and seed production of the plants have to be taken into consideration (Baldwin, 1998; Kruidhof et al., 2012). Further investigation on the impact of the artificial application of methyl jasmonate and *cis*-jasmone on the plants inducing defence mechanisms and on other arthropod species will be necessary to evaluate the potential of the jasmonates for biological control of western flower thrips (Farmer & Ryan, 1990; Browse & Howe, 2008).
